# Sodium Tanshinone IIA Silate Exerts Microcirculation Protective Effects against Spinal Cord Injury In Vitro and In Vivo

**DOI:** 10.1155/2020/3949575

**Published:** 2020-10-08

**Authors:** Xing Li, Dan Luo, Yu Hou, Yonghui Hou, Shudong Chen, Jiheng Zhan, Jiyao Luan, Le Wang, Dingkun Lin

**Affiliations:** ^1^Department of Orthopedic Surgery, The Second Affiliated Hospital of Guangzhou University of Chinese Medicine, No. 111 Dade Road, Guangzhou, Guangdong 510120, China; ^2^Guangzhou University of Chinese Medicine, No. 12, Jichang Road, Baiyun District, Guangzhou 510405, China; ^3^Lingnan Medical Research Center of Guangzhou University of Chinese Medicine, Guangzhou 510405, China; ^4^Department of Spine Surgery, The First Affiliated Hospital of Sun Yat-sen University, Guangzhou, China

## Abstract

Spinal cord microcirculation involves functioning endothelial cells at the blood spinal cord barrier (BSCB) and maintains normal functioning of spinal cord neurons, axons, and glial cells. Protection of both the function and integrity of endothelial cells as well as the prevention of BSCB disruption may be a strong strategy for the treatment of spinal cord injury (SCI) cases. Sodium Tanshinone IIA silate (STS) is used for the treatment of coronary heart disease and improves microcirculation. Whether STS exhibits protective effects for SCI microcirculation is not yet clear. The purpose of this study is to investigate the protective effects of STS on oxygen-glucose deprivation- (OGD-) induced injury of spinal cord endothelial cells (SCMECs) *in vitro* and to explore effects on BSCB and neurovascular protection *in vivo*. SCMECs were treated with various concentrations of STS (1 *μ*M, 3 *μ*M, and 10 *μ*M) for 24 h with or without OGD-induction. Cell viability, tube formation, migration, and expression of Notch signaling pathway components were evaluated. Histopathological evaluation (H&E), Nissl staining, BSCB permeability, and the expression levels of von Willebrand Factor (vWF), CD31, NeuN, and Notch signaling pathway components were analyzed. STS was found to improve SCMEC functions and reduce inflammatory mediators after OGD. STS also relieved histopathological damage, increased zonula occludens-1 (ZO-1), inhibited BSCB permeability, rescued microvessels, protected motor neuromas, and improved functional recovery in a SCI model. Moreover, we uncovered that the Notch signaling pathway plays an important role during these processes. These results indicated that STS protects microcirculation in SCI, which may be used as a therapeutic strategy for SCI in the future.

## 1. Introduction

Spinal cord injury (SCI) is a major medical issue that can lead to permanent paraplegia [1]. Only 14% of nurses and 37% of physicians take effective resuscitative measures following SCI cases [2]. SCI not only influences both the mental and physical health of the patient but also influences treatment [3–5]. Although various therapies including surgery and drugs are used for SCI, there is currently no cure for this injury [6–8].

Spinal cord microcirculation plays an important role in maintaining normal function of spinal cord neurons, axons, and glial cells [9, 10]. At the primary mechanical injury stage, compression damages pericytes and endothelial cells, fractures blood vessels, and disturbs the blood spinal cord barrier (BSCB), which results in vascular imbalances and leads to severe secondary injury and functional disability [11, 12]. BSCB functions similarly to the blood-brain barrier (BBB), which is based on the integrity of endothelial cells and their accessory structures. After SCI, BSCB breaks down and neurotoxic products and immune cells infiltrate into the injured parenchyma, which contributes to secondary damage [13–15]. These secondary injuries result in the death of neurons and glia and permanent neurological disability [15, 16]. Therefore, protecting the function and integrity of endothelial cells and preventing BSCB disruption are necessary to decrease severe secondary injury, which may serve as a potential therapeutic strategy for SCI.

**Figure 1:**
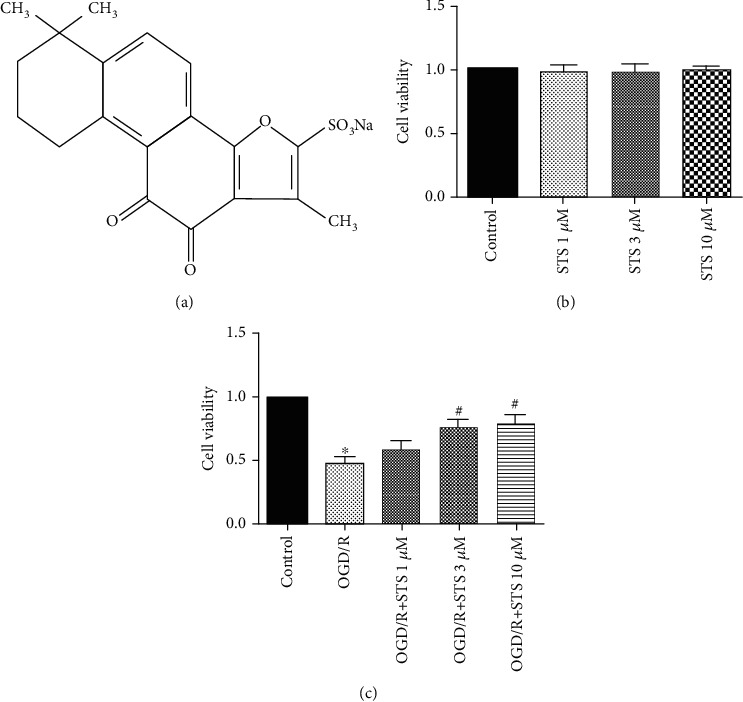
STS improves SCMEC survival under OGD conditions. (a) The chemical structure of STS. (b) SCMECs were treated with STS at various concentrations (1 *μ*M, 3 *μ*M, and 10 *μ*M) for 24 h. (c) SCMECs were cultured with STS (3 *μ*M) after OGD for 24 h. Data are presented as the mean ± S.D. ^∗^*P* < 0.05 vs. the control group. ^#^*P* < 0.05 vs. the OGD group.

Danshen derived from rhizome of Salviae miltiorrhizae Bge or dried root is a major component of traditional Chinese medicinal herbs [17]. Tanshinone IIA is the most active diterpenoid quinine pigment in Danshen. Many studies have shown that Tanshinone IIA has a medicinal effect on cardiovascular, cerebrovascular, endocrine, and nervous system diseases [18–21]. Sodium Tanshinone IIA silate (STS, structure shown in Figure 1(a)) is a water-soluble derivative of Tanshinone IIA [22]. STS is used to treat coronary heart diseases in China. Some research also indicated that STS protects against inflammation, oxidative stress, and cell death [23–26]. However, the mechanisms behind Tanshinone IIA influencing STS-mediated protective effects after SCI microcirculation are still unclear.

In this study, we investigated the protective effects of STS on oxygen-glucose deprivation- (OGD-) induced injury of spinal cord endothelial cells *in vitro* and explored improvements of the BSCB and neurovascular protection *in vivo*.

## 2. Materials and Methods

### 2.1. Materials

Healthy adult male C57BL/6 mice were purchased from the Guangzhou University of Chinese Medicine (Guangzhou, China). All studies were performed according to the guidelines provided by Guangzhou University of Chinese Medicine. Research was approved by the ethics committee of Guangzhou University of Chinese Medicine. Endothelial growth medium 2 (EGM-2) was obtained from LONZA, (California, USA). DMEM, fetal bovine serum (FBS), trypsin, and penicillin/streptomycin were obtained from Gibco (NY, USA). The WST-1 cell viability detection kit was obtained from Nanjing KeyGEN Biotech Co., Ltd. (Nanjing, China). STS (purity > 99% using HPLC) was purchased from Chengdu Herbpurify Co., Ltd. (Chengdu, China), dissolved in sterile water (St. Louis, MO, USA), and diluted in medium.

### 2.2. Endothelial Cell Isolation

Spinal cord microvascular endothelial cells (SCMECs) were isolated as previously described by collecting microvessels from 10 spinal cords of 4-5-week-old mice [27, 28]. Endothelial cells were plated into culture dishes containing EGM-2 supplemented with 5% fetal bovine serum, 1% penicillin, and 1% streptomycin. Plates were incubated at 37°C (5% CO_2_). Medium was replaced once every two days. Endothelial cells with lower passages (3 to 5) were used in all experiments.

### 2.3. Immunofluorescence Analysis

SCMECs were fixed in 4% paraformaldehyde, permeabilized with 0.3% Triton X-100, and blocked in 5% normal goat serum diluted in PBS. After an overnight incubation with primary antibodies against vWF (1: 200, Santa Cruz Biotechnology), CD31 (1: 200, Abcam), NG2 (1: 200, Abcam), and GFAP (1: 200, Abcam) at 4°C, cells were washed three times in PBS for 5 min and subsequently incubated with fluorescein-conjugated secondary antibodies. Nuclei were stained with DAPI. Fluorescence microscopy (Olympus, Japan) was used to analyze results.

### 2.4. *In Vitro* OGD Model

To mimic SCI conditions *in vitro*, the OGD model was used [29, 30]. SCMECs were washed in PBS and replaced by glucose-free medium (RPMI-1640 without FBS). SCMECs were then transferred to a hypoxia chamber (BioSpherix, Lacona, NY), which contained 94% N_2_, 5% CO_2_, and 1% O_2_ for 7 hours. To terminate OGD, SCMECs were placed in medium (EGM-2 with 5% FBS, 1% streptomycin and 1% penicillin) including STS at the different concentrations (1 *μ*M, 3 *μ*M, and 10 *μ*M) and incubated in a 37°C, 5% CO_2_ incubator. SCMECs without exposure to OGD were used as controls. RO4929097 (1 *μ*M) was used as an inhibitor of the Notch signaling pathway.

### 2.5. Cell Viability

Cell viability was measured using the WST-1 assay [31]. Briefly, SCMECs were added to 96-well plates at a density of 1 × 10^4^/well. SCMECs were treated with different concentrations of STS (1 *μ*M, 3 *μ*M, and 10 *μ*M) for 24 h or treated with various concentrations of STS (1 *μ*M, 3 *μ*M, and 10 *μ*M) for 24 h after OGD. After incubation, 10 *μ*l of WST-1 solution was added to the plate for 2 h at 37°C. A microplate reader (Bio-Rad, USA) was used to analyze the absorbance at a wavelength of 450 nm. All experiments were performed in biological triplicate.

### 2.6. Tube Formation

SCMECs were seeded on a Matrigel basement membrane matrix (BD, CA, USA) to examine tube formation. Matrigel (50 *μ*l) was added to the center of each well of a 96-well plate. The plate was then allowed to solidify for 30 min at 37°C. After exposure to OGD and incubation with STS for 24 h, 1.5 × 10^4^ cells/well were plated to form tubes. Cells were allowed to incubate for an appropriate period of time at 37°C (5% CO_2_). Photographs were acquired using an inverted microscope (200x). The number of junctions and the total length of tubes were counted using ImageJ.

### 2.7. Migration Assay

The migration of SCMECs was evaluated using transwell insert chambers (BD, USA) containing a polycarbonate filter membrane with an 8 *μ*m pore size. After exposure to OGD and incubation with STS for 24 h, cell density was adjusted to 5 × 10^5^ cells/ml in EGM-2 supplemented with 1% FBS. For the transwell assay, 200 *μ*l of cells was seeded into the upper chamber, where EGM-2 supplemented with 5% FBS was added to the lower chamber. After a 24 h incubation period, migrated cells were fixed in paraformaldehyde for 30 min and stained with crystal violet for 4 h. Cells in the upper chamber were removed using a cotton-tipped swab. Photographs were acquired using optical microscopy (100x), and migrated cells were counted and averaged in five random visual fields.

### 2.8. Scratch Wound Healing

SCMECs were added into 6-well plates at a density of 5 × 10^5^ cells per well. After exposure to OGD and incubation with STS for 24 h, a scratch wound was gently created using a 200 *μ*l sterile pipette tip on a uniform layer of cells. Cells were also rinsed with PBS to remove debris and floating cells. Wound healing was photographed at 0, 12, and 24 h in the same area using an inverted microscope. All experiments were performed in biological triplicate.

### 2.9. Determination of Inflammatory Mediators

According to instructions provided by the manufacturer, the concentrations of IL-6, TNF-*α*, and IL-1*β* in culture media were measured using commercially available ELISA kits (Nanjing KeyGEN Biotech Co., Ltd., Nanjing, China).

### 2.10. Animal Care and Establishment of SCI Model and Experiment Groups

Healthy adult male C57BL/6 mice were obtained from the Guangdong Medical Experimental Animal Center (Foshan, China, Certificate No.44007200047868) and housed in strictly controlled environmental conditions with 12 : 12 light/dark cycles at 25 ± 1°C and 55 ± 5% relative humidity and free access to food and water. All animal experiments were approved by the Institutional Animal Care and Use Committee of Guangzhou University of Chinese Medicine and performed according to the “NIH Guide for the Care and Use of Laboratory Animals”.

A total of 30 adult male C57BL/6 mice (20-25 g) were randomly divided into 3 groups (*n* = 10 per group) including the sham-operated, SCI model, and STS-treated groups. Allen's method was used to establish the SCI model with a moderate contusion, based on previous methods [32]. Mice were anesthetized using pentobarbital sodium (30 mg/kg, i.p.). Spinal cords were exposed through T9-T10 laminectomy under sterile conditions followed by a contusion with an impact velocity of 0.5 m/s, depth of 0.6 mm, and duration of 80 ms. Successful establishment of the SCI model was observed by spinal cord congestion, leg swaying, tail swing reflexes, and slow paralysis. Next, the bladder was manually pressed twice a day. STS (dissolve in saline, 20 mg/kg) was administered through intraperitoneal injection at 2 h after SCI once a day for a week. Similar methodology was applied to the vehicle groups. After treatment, mice were sacrificed using pentobarbital sodium (80 mg/kg, i.p.), and spinal cords (5 mm sections of the spinal cord centered at the lesion site) were extracted and stored at -80°C for immunofluorescence and western blotting experiments or were perfused with paraformaldehyde for histopathological evaluation (H&E) and Nissl staining. The detailed experimental design for this study is shown in supplementary materials (Figure 1S).

### 2.11. Functional Scale

The Basso, Beattie, and Bresnahan (BBB) scale, which measures locomotor ability for 4 min according to the 21 different criteria for movement of the hindlimb, was used to evaluate the functional recovery at 1, 3, 5, 7, 14, 21, and 28 days post-SCI [33, 34]. Two trained investigators blinded to the experimental conditions analyzed the scale, and final scores were averaged for each group.

### 2.12. H&E and Nissl Staining

Paraffin sections (5 *μ*m thickness) were deparaffinized using xylene and underwent H&E and Nissl staining based on instructions provided by the manufacturer [35]. Photographs were taken using a light microscope (Leica, Germany). Injured neurons were counted, and the histopathological alteration of gray matter was scored on a 6-point scale for H&E staining where 0 = no observable lesions, 1 = gray matter containing 5-10 eosinophilic neurons, 3 = gray matter containing >10 eosinophilic neurons, 4 ≤ 1/3 of gray matter area infarction, 5 = 1/3-1/2 of gray matter area infarction, and 6 ≥ 1/2 of gray matter area infarction [36]. All histological examinations were performed blindly.

Rexed's lamina system of gray matter was used to classify and count neurons for Nissl staining [37]. The pathological score was calculated as the average of all sections from a single spinal cord for each animal. All histological examinations were performed blindly.

### 2.13. BSCB Permeability

Evans Blue dye (Aladdin, China) leakage was evaluated to analyze the permeability of the BSCB [38, 39]. A total of 2 ml of 2% Evans Blue dye mixed in saline was administered by intravenous injection at 7 days after SCI. Three hours later, mice were anesthetized and perfused with PBS and 4% paraformaldehyde intracardiacally until Evans Blue dye did not run out of the right atrium. To qualitatively examine Evans Blue extravasation, photographs of gross view changes were taken to observe whole spinal cords. Moreover, paraformaldehyde-fixed spinal cords were sectioned at a thickness of 20 *μ*m. The relative fluorescence intensity of Evans Blue was measured using fluorescent microscopy (Olympus, Japan). The relative fluorescence intensity was quantified using ImageJ.

### 2.14. Immunofluorescence

Ten-micrometer-thick transverse frozen slices were heated at room temperature for 30 min, permeabilized using 0.3% Triton X-100, and blocked in 5% normal goat serum diluted in PBS for 1 hour. Next, slices were incubated overnight at 4°C in primary antibodies targeting vWF (1 : 200, Santa Cruz Biotechnology) or NeuN (1 : 300, Abcam). Spinal cords were subsequently incubated in fluorescein-conjugated secondary antibodies for 1 h at room temperature. Nuclei were stained using DAPI, and fluorescence microscopy (Olympus, Japan) was used to analyzed results.

### 2.15. Western Blotting

Western blotting experiments were performed according to previous studies [40]. Briefly, proteins (50 *μ*g) were loaded into a 10% SDS-PAGE gel and electrotransferred to a polyvinylidene difluoride membrane (Millipore, USA). Membranes were incubated overnight at 4°C in primary antibodies recognizing Jagged-1 (1 : 1000, Cell Signaling Technology), Notch-1 (1 : 1000, Cell Signaling Technology), Hes-1 (1 : 1000, Cell Signaling Technology), CD31 (1 : 1000 dilution, Abcam), zonula occludens-1 (ZO-1) (1 : 1000 dilution, Abcam), and vWF (1 : 500, Santa Cruz Biotechnology). Membranes were washed in TBS and incubated in secondary antibodies conjugated to horseradish peroxidase (HRP) (1 : 1000, Cell Signaling Technology) for 2 h at room temperature. GAPDH (1 : 1000 dilution, Abcam) was used as an endogenous loading control. The ImageQuant LAS 4000 mini detection system (GE Healthcare, Buckinghamshire, UK) was used for quantified densitometric analysis, and ImageJ software (National Institutes of Health, Bethesda, MD) was used to analyze results.

### 2.16. Statistical Analysis

All data were presented as the mean ± standard deviation. Statistical analyses were performed using SPSS 24.0 software (SPSS Inc., USA). Student's *t*-test (normal distribution) or Mann–Whitney *U* test (nonnormal distribution) was used to identify differences between two groups, and one-way analysis of variance (normal distribution) or Kruskal–Wallis (non-normal distribution) test followed by Bonferroni or Dunn post hoc test was performed to compare three or more groups. *P* values less than 0.05 were considered statistically significant.

## 3. Results

### 3.1. Characterization of SCMECs

**Figure 2:**
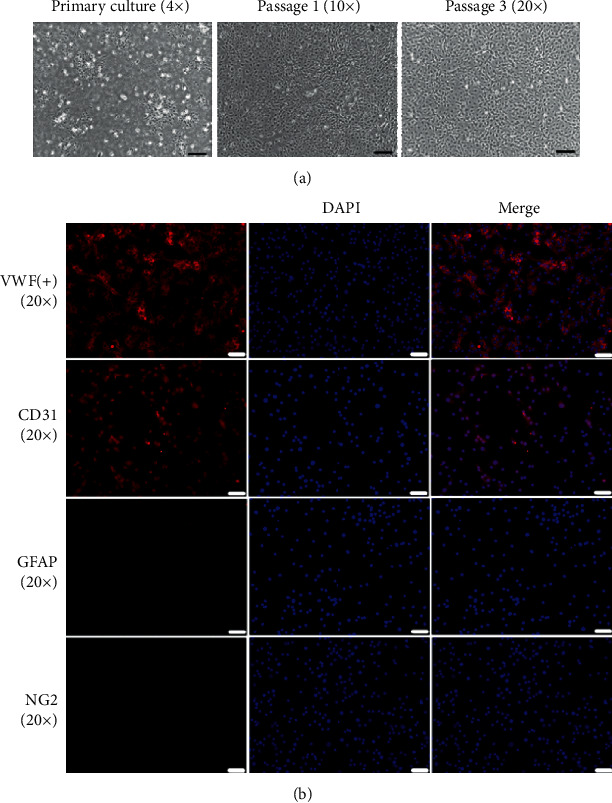
Morphological characterization and immunofluorescence analysis of SCMECs. (a) Representative fields of SCMEC morphologies at the primary, first, and third passages (scale bar = 200 *μ*m, 100 *μ*m, and 50 *μ*m). (b) Representative immunofluorescent labeling images for CD31, vWF, NG-2, and GFAP (scale bar = 50 *μ*m).

Proliferation of SCMECs isolated from vessels is depicted in Figure 2(a). Immunofluorescence analysis revealed that SCMEC markers CD31 and vWF were expressed. However, the pericyte marker NG-2 and the astrocyte maker GFAP were not expressed (Figure 2(b)). These results indicated that there were no pericytes or astrocytes mixed in SCMEC cultures.

### 3.2. STS Improves the Survival of SCMECs under OGD

First, the cytotoxicity of SCMECs treated with STS at various concentrations (1 *μ*M, 3 *μ*M, and 10 *μ*M) for 24 h was measured using the WST-1 assay. As shown in Figure 1(b), there was no significant increase in cytotoxicity for SCMECs treated with STS compared to the control group. We then examined the protective effects of STS on SCMECs exposed to OGD. STS (3 *μ*M) significantly improved SCMEC survival rate (Figure 1(c)) compared to the OGD group.

### 3.3. STS Promotes Tube Formation in SCMECs after OGD

**Figure 3:**
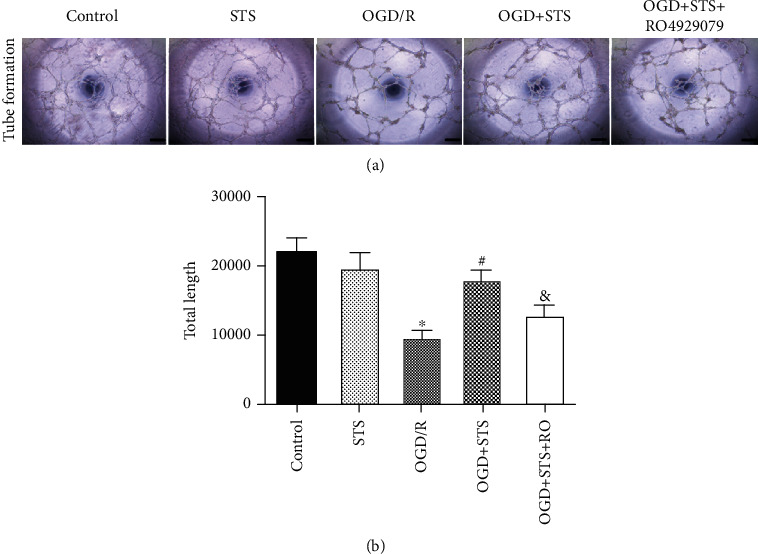
STS promotes tube formation in SCMECs after OGD. SCMECs were cultured with STS (3 *μ*M) after OGD for 24 h. (a) A tube formation assay was used to evaluate tube formation. (b) Bar graphs show the count of total tube length. Data are presented as the mean ± S.D. ^∗^*P* < 0.05 vs. the control group, ^#^*P* < 0.05 vs. the OGD group, and ^&^*P* < 0.05 vs. the OGD+STS group. Scale bar = 200 *μ*m.

A Matrigel angiogenesis (tube formation capacity) assay was used to illustrate whether STS promotes tube formation in SCMECs after OGD. There was a reduction in the number of tube-like structures after OGD, where STS treatment effectively promoted tube formation. RO4929097, a notch signaling inhibitor, significantly blocked the protective effects of STS on tube formation (Figure 3(a)).

### 3.4. STS Enhances the Migration of SCMECs Exposed to OGD

**Figure 4:**
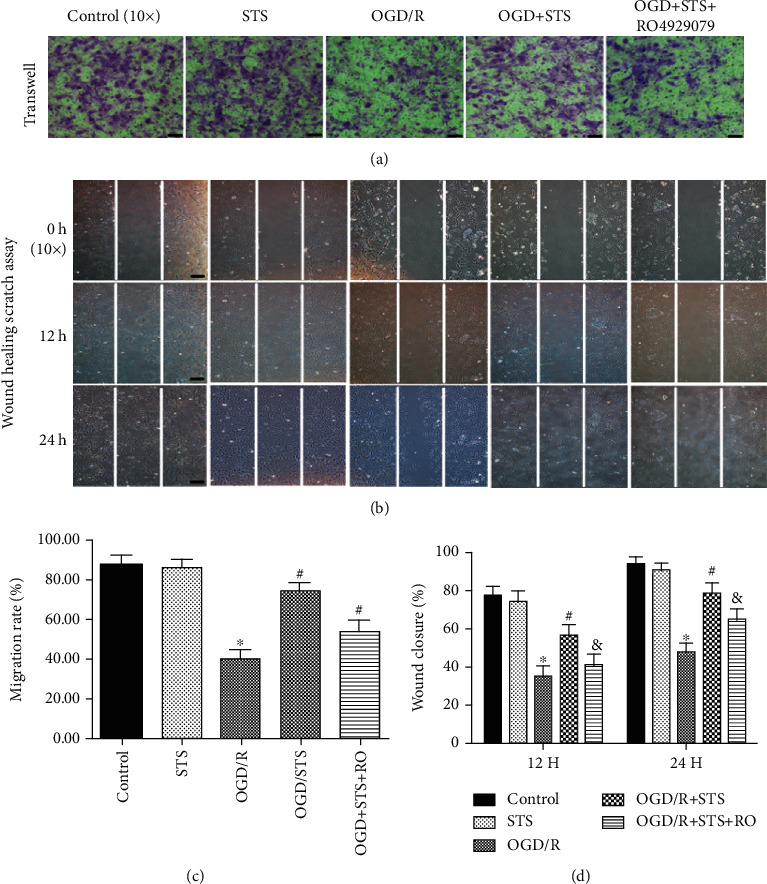
STS enhances migration of SCMECs exposed to OGD. SCMECs were cultured with STS (3 *μ*M) after OGD for 24 h. (a) The influence of STS on migration after OGD was analyzed by a transwell assay (scale bar = 100 *μ*m). (b) Wound healing scratch assay revealing the influence of STS on migration at 0, 12, and 24 h (scale bar = 100 *μ*m). (c) Quantitative analysis of the migration rate. (d) Bar graphs depicted the migration rate of wound closure. Data are presented as the mean ± S.D. ^∗^*P* < 0.05 vs. the control group, ^#^*P* < 0.05 vs. the OGD group, and ^&^*P* < 0.05 vs. the OGD+STS group.

Migration is an important step for the angiogenesis of endothelial cells. Wound healing scratch and transwell assays were used to evaluate the migration of SCMECs treated with STS after OGD. In the transwell assay, the number of migrating SCMECs significantly decreased after OGD, where treatment with STS increased migration compared to the OGD group (Figure 3(b)). Similar results were also observed for wound healing scratch assays. As shown in Figure 4, SCMECs treated with STS quickly migrated into the injured area after 12 h and 24 h following the scratch compared to the OGD group. Furthermore, RO4929097 partially blocked the migration of SCMECs treated with STS as observed in both the wound healing scratch and transwell assays.

### 3.5. STS Reduces Inflammatory Mediators of SCMECs after OGD

**Figure 5:**
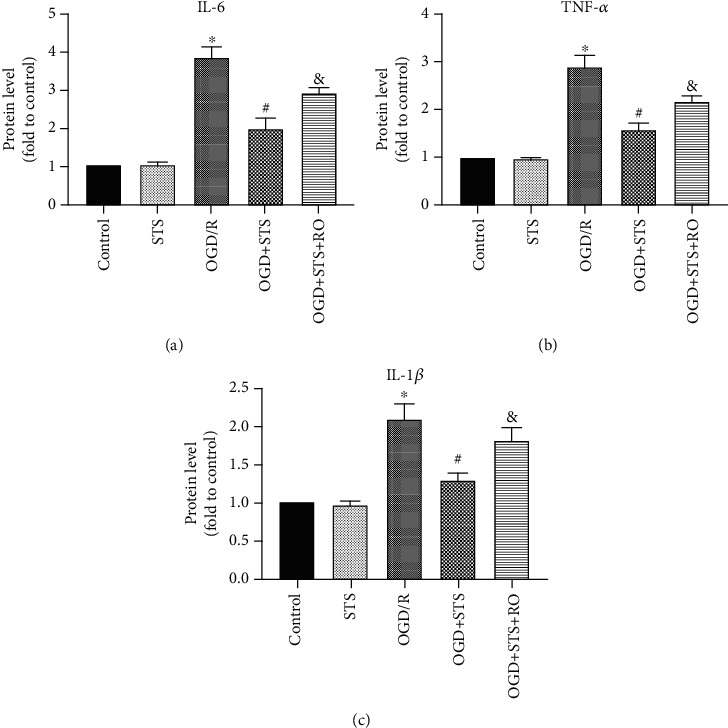
STS reduces inflammatory mediators on SCMECs after OGD. The culture medium of SCMECs was collected with STS (3 *μ*M) after OGD for 24 h. Levels of IL-6 (a), TNF-*α* (b), and IL-1*β* (c). Data are presented as the mean ± S.D. ^∗^*P* < 0.05 vs. the control group, ^#^*P* < 0.05 vs. the OGD group, and ^&^*P* < 0.05 vs. the OGD+STS group.

To explore the anti-inflammatory effects of STS against OGD in SCMECs, the concentrations of IL-6, TNF-*α*, and IL-1*β* in culture media were measured. As shown in Figure 5, compared to the OGD group, the expression levels of IL-6, TNF-*α*, and IL-1*β* were significantly decreased after treatment with STS, and RO4929097 blocked these effects. These results indicate that STS exerts anti-inflammatory functions against OGD in SCMECs.

### 3.6. STS Exerts Protective Effects on SCMECs after OGD through Notch Signaling

**Figure 6:**
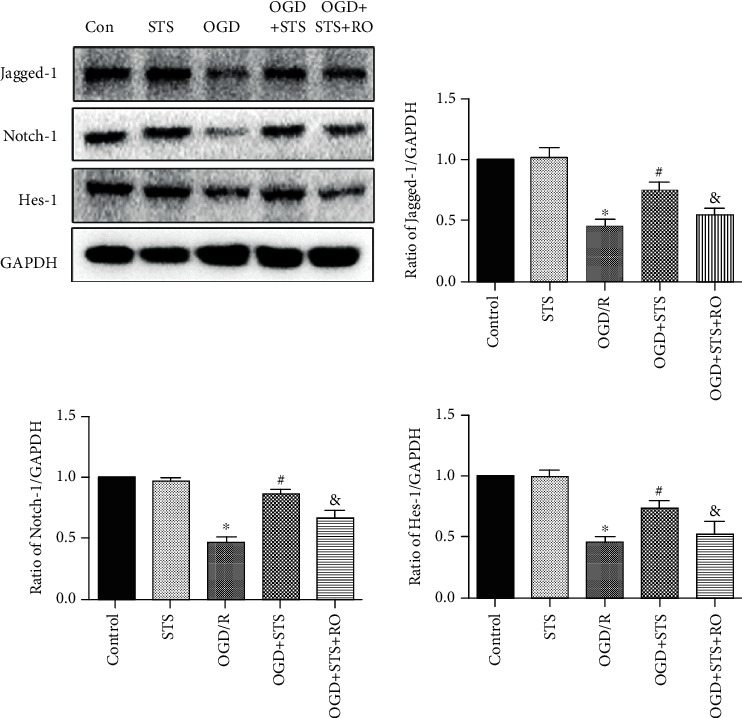
STS exerts protective effects on SCMECs after OGD through the Notch signaling pathway. SCMECs were cultured with STS (3 *μ*M) after OGD for 24 h. Western blotting was used to measure protein expression of Jagged-1, Hes-1, and Notch-1. Data are presented as the mean ± S.D. ^∗^*P* < 0.05 vs. the control group, ^#^*P* < 0.05 vs. the OGD group, and ^&^*P* < 0.05 vs. the OGD+STS group.

To investigate whether the Notch signaling pathway is involved in the protective effects of STS against OGD in SCMECs, the expression levels of Jagged-1, Notch-1, and Hes-1 proteins were analyzed. Expression levels of Jagged-1, Hes-1, and Notch-1 were significantly decreased in the OGD group. However, this effect was significantly reversed when treated with STS (Figure 6). Moreover, RO4929097 blocked protective effects observed against OGD when SCMECs were treated with STS.

### 3.7. STS Relieves Histopathological Damage and Improves Functional Recovery after SCI

**Figure 7:**
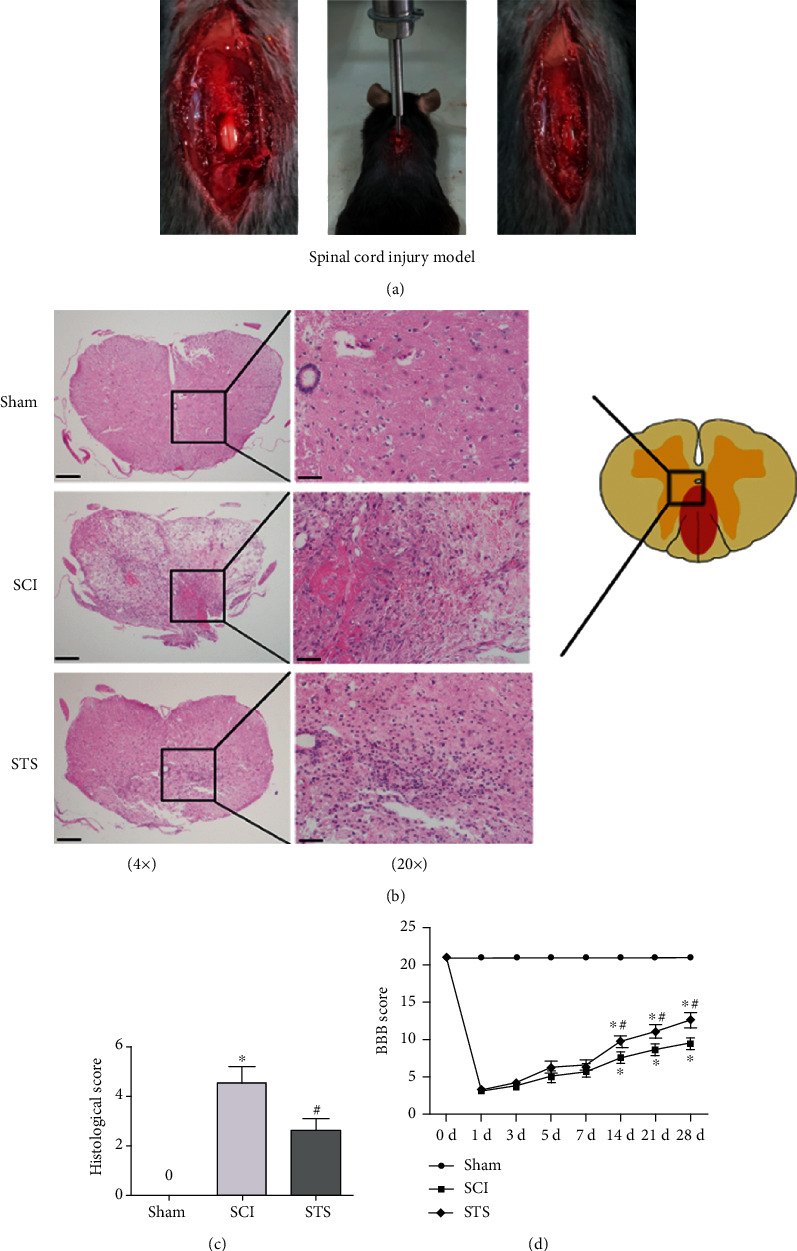
STS relieves histopathological damage after SCI. (a) Establishment of the SCI model. (b) Representative H&E staining 7 days after SCI (scale bar = 200 *μ*m and 50 *μ*m). (c) Quantitative analysis of H&E staining. (d) BBB score was used to evaluate functional recovery at 1, 3, 5, 7, 14, 21, and 28 days after SCI. Data are presented as the mean ± S.D. ^∗^*P* < 0.05 vs. the control group; ^#^*P* < 0.05 vs. the SCI group (*n* = 6).

To explore the protective effects of STS on histopathological injury, H&E staining was used to evaluate histopathological alterations. Staining (Figure 7(b)) revealed significant histopathological changes after SCI (4.31 ± 0.63) compared to the sham group. These histopathological changes included diffuse hemorrhage, widespread edema, congestion, neutrophil infiltration, and neuronal disruption. However, after STS treatment, these effects were attenuated (2.23 ± 0.49). To evaluate functional recovery, the BBB score was used. As shown in Figure 7(d), compared to the SCI group, BBB scores were significantly increased in the SCI+STS groups at 14, 21, and 28 days. These results indicate that STS relieves histopathological damage and improves functional recovery after SCI.

### 3.8. STS Inhibits BSCB Permeability and Increases Tight Junction Proteins after SCI

**Figure 8:**
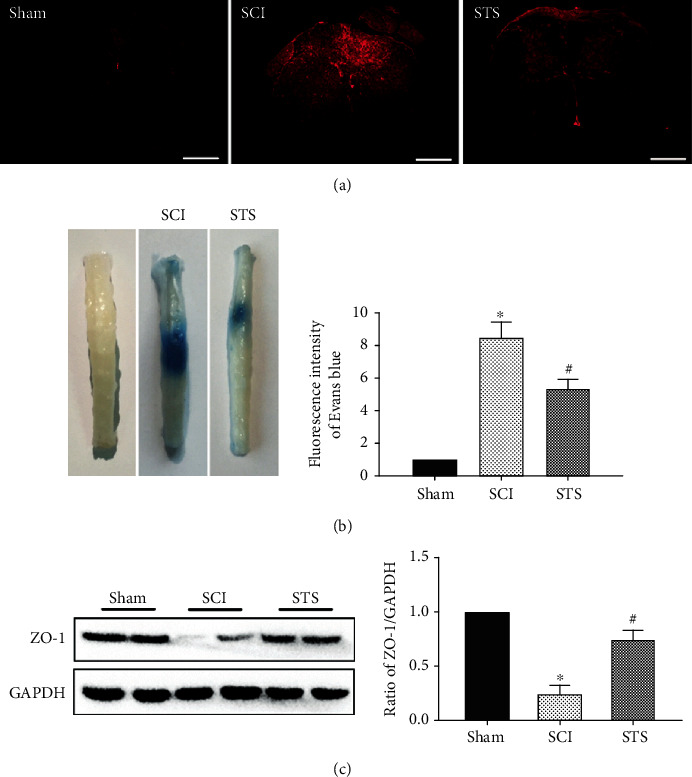
STS inhibits BSCB permeability after SCI. (a) Photographs of whole spinal cords demonstrating Evans Blue dye extravasation into the spinal cord 7 days after injury for sham, SCI, and STS groups. (b) Immunofluorescence labeling of Evans Blue dye extravasation was analyzed in the sham, SCI, and STS groups. (c) Western blotting was used to measure protein expression of ZO-1. Data are presented as the mean ± S.D. ^∗^*P* < 0.05 vs. the control group; ^#^*P* < 0.05 vs. the SCI group (*n* = 6). Scale bar = 200 *μ*m.

Evans Blue assay was used to determine how STS influences BSCB permeability 7 days after injury. As shown in Figure 8(a), the permeated area was higher in the SCI group compared to the sham group. After treatment with STS, the permeated area was significantly inhibited. Meanwhile, the fluorescence intensity of Evans Blue dye extravasation was also evaluated to show that STS treatment significantly reduced fluorescent intensity of dye extravasation at 7 days after injury compared to the SCI group (Figures 8(b) and 8(c)).

Tight junction proteins are vital structural proteins in the BSCB. To further evaluate the protective effects of STS on BSCB, the expression of ZO-1 was analyzed by western blotting. As shown in Figure 8(d), compared to the SCI group, the expression of ZO-1 was significantly increased in the SCI+STS groups. These results illustrated that STS prevented BSCB disruption and protected the integrity of BSCB function after SCI.

### 3.9. STS Rescues Microvessels after SCI

**Figure 9:**
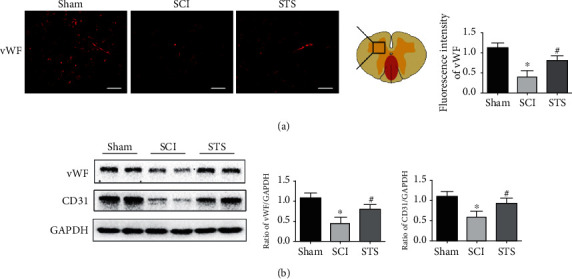
STS rescues microvessels after SCI. Microvessels were evaluated for the expression of vWF and CD31 proteins. (a) Immunofluorescence labeling of vWF was measured 7 days after injury in the sham, SCI, and STS groups. (b) Immunoblotting was used to detect the expression levels of vWF and CD31 proteins. Data are presented as mean ± S.D. ^∗^*P* < 0.05 vs. the control group; ^#^*P* < 0.05 vs. the SCI group (*n* = 6). Scale bar = 50 *μ*m.

The endothelial marker proteins vWF and CD31 were analyzed by immunofluorescence and western blotting. As shown in Figure 9(a), the amount of fluorescence intensity labeled by a vWF antibody was lower in the SCI group compared to the sham group. A significant increase in the proportion of vWF-labeled blood vessels was observed after treatment with STS. Moreover, STS treatment significantly increased the expression of vWF and CD31 proteins 7 days after injury compared to the SCI group (Figure 9(b)).

### 3.10. STS Protects Motor Neurons after SCI

**Figure 10:**
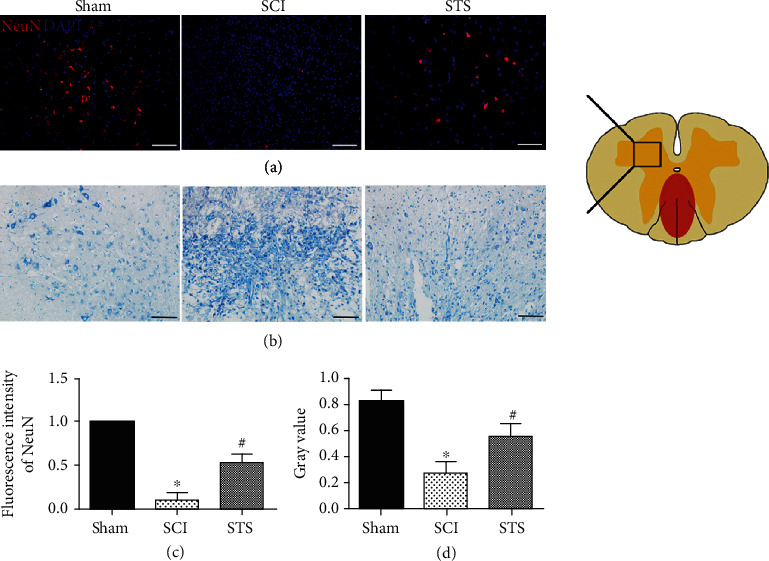
STS protects motor neurons after SCI. (a) Immunofluorescence labeling of NeuN was detected 7 days after SCI. (b) Representative Nissl staining 7 days after SCI. (c) Quantitative analysis of fluorescence intensity. (d) Quantitative analysis of Nissl staining. Data are presented as the mean ± S.D. ^∗^*P* < 0.05 compared to the control group; ^#^*P* < 0.05 compared with the SCI group (*n* = 6). Scale bar = 50 *μ*m.

To confirm whether there was a neuroprotective effect of STS after SCI, we evaluated the neuronal marker NeuN and Nissl staining 7 days after injury. As shown in Figure 10(a), a lower number of motor neurons were observed in the SCI group compared to the sham group. After treatment with STS, the number of motor neurons was significantly increased. The expression of Nissl bodies was reduced in the SCI group (sham (0.82 ± 0.08) versus SCI (0.33 ± 0.10)), where results were reversed with STS treatment (STS (0.59 ± 0.12) versus SCI (0.33 ± 0.10)) (Figure 10(b)).

### 3.11. STS Exerts Microcirculation Protective and Neuroprotective Effects after SCI through Notch Signaling

**Figure 11:**
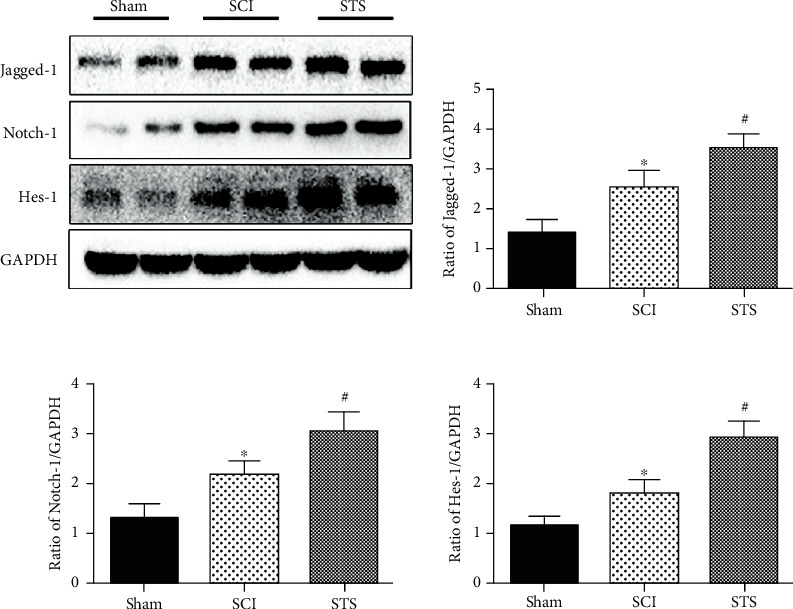
STS exerts microcirculation protective and neuroprotective effects after SCI through the Notch signaling pathway. Western blotting was used to detect the expression levels of Jagged-1, Hes-1, and Notch-1 proteins in spinal tissues 3 days after SCI. Data are presented as the mean ± S.D. ^∗^*P* < 0.05 vs. the control group; ^#^*P* < 0.05 vs. the SCI group (*n* = 6).

To confirm whether the STS microcirculation protective and neuroprotective effects after SCI were activated through Notch signaling, the expression levels of Jagged-1, Hes-1, and Notch-1 in spinal tissues were analyzed 3 days after injury. As shown in Figure 11, protein expression of Jagged-1, Hes-1, and Notch-1 was significantly reduced in the SCI group. After treatment with STS, this effect was significantly reversed.

## 4. Discussion

SCI leads to irreversible neurological injury that affects 180,000 patients worldwide every year [41]. There is a greater number of patients with SCI in China compared to other countries [42]. An initial mechanical contusion is the main cause of SCI, which results in hemorrhages, ischemia, and disorders of microhemodynamics as well as destruction of the BBB [43–46]. Moreover, spinal cord microcirculation plays an important role in maintaining the normal function of spinal cord neurons, axons, and glial cells [9, 10]. Spinal cord microcirculation is also a key factor in progressive degeneration and subsequent functional injuries after SCI [45, 47, 48].

Sodium Tanshinone IIA silate (STS) is a water-soluble derivative of Tanshinone IIA that plays a role in microcirculation. Many studies have shown that Tanshinone IIA exhibits antiangiogenic effects in normal or overgrown conditions, such as tumors. Lee et al. [49] reported that Tanshinone IIA inhibited angiogenesis in VEGF-induced tube formation in human EPCs. Tsai et al. [50] demonstrated that Tanshinone IIA inhibited cancer development and tumor angiogenesis through antiangiogenic effects. However, in response to ischemia-reperfusion injury, Tanshinone IIA improves postischemic angiogenesis and microcirculation and promotes recovery [51–53].

In this study, we found that STS treatment improves SCMEC functions after OGD *in vitro*. Under OGD conditions, treatment with STS significantly reduced inflammatory mediators (IL-6, TNF-*α*, and IL-1*β*), improved survival, promoted tube formation, and enhanced the migration ability of SCMECs. Meanwhile, similar protective microcirculation effects were demonstrated after treatment with STS in an *in vivo* SCI model. After SCI and treatment with STS, histopathological damage was relieved, BSCB permeability was inhibited, microvessels were rescued, motor neurons were protected, and functional recovery was improved. Moreover, the Notch signaling pathway was found to be involved in these processes both *in vitro* and *in vivo*. This is the first study illustrating that STS has protective effects against SCI through the Notch signaling pathway.

The OGD model mimics ischemia *in vitro* and is widely applied for studying brain and spinal cord injuries [54–56]. Thus, the OGD model was used in this study to determine whether STS could protect SCMEC functions critical for SCI recovery. We showed that OGD significantly decreased viability and functionality of SCMECs, which is consistent with other findings [28, 57–59]. However, STS treatment reverses these effects and indicates that STS protects SCMEC functions. Meanwhile, many studies reported that inflammation was the second major component impacting SCMEC functions and BSCB integrity [60, 61]. Inflammatory mediators including IL-6, TNF-*α*, and IL-1*β* can recruit inflammatory cells to invade the central nervous system through a damaged BSCB. Therefore, we measured inflammatory mediators (IL-6, TNF-*α*, and IL-1*β*) *in vitro*, and the results showed that the expression levels of IL-6, TNF-*α*, and IL-1*β* were significantly decreased when exposed to STS.

The blood vessel-specific markers CD31 and vWF reflect vascular structure and function [38, 39]. After SCI, spinal cord repair and remodeling require blood vessels to supply nutrition and oxygen [62–64]. Consistent with previous findings [38, 39], a decrease in blood vessel area and lower levels of CD31 and vWF were observed in SCI mice. STS treatment significantly increased the blood vessel area as well as CD31 and vWF protein levels. These findings illustrated that STS improves angiogenesis.

The BSCB limits the entry of blood cells and plasma components into the spinal cord to protect the central nervous system [65, 66]. When the BSCB is disrupted, neurotoxic products and immune cells infiltrate into the injured parenchyma contributing to secondary damage [13–15]. Hence, we evaluated the BSCB in SCI mice treated with STS or vehicle. The permeated Evans Blue area from the BSCB was greater after SCI, as shown previously [38, 39, 67, 68]. Moreover, this was reversed with STS treatment. Similar results were shown by fluorescence intensity of Evans Blue dye extravasation. Tight junction proteins are located around the interendothelial space, sealing the BSCB [69]. Disruption of tight junction proteins may lead to BSCB permeability during SCI [60]. ZO-1 is a cytoplasmic tight junction protein that plays an important role in tight junction protein maintenance and formation [70]. In this study, ZO-1 expression was significantly increased after STS treatment. Altogether, this indicated that STS protects BSCB integrity, which ultimately protects the central nervous system after SCI.

Furthermore, neuroprotective effects of STS treatment in SCI mice were measured by analyzing the expression of NeuN proteins and Nissl bodies in spinal tissues. We found that NeuN-positive neuronal cells and Nissl bodies were increased after STS treatment, which was consistent with a greater blood vessel area with STS treatment as shown previously. These findings provide evidence that an increase in blood vessels has a beneficial effect on neuron survival.

The Notch signaling pathway is critical for cell differentiation, proliferation, and apoptosis [71–73]. There are four Notch receptors (Notch1-4) that can be activated by a membrane-bound ligand (Jagged-1,2/delta-like-1,3,4). When the pathway is activated, the Notch intracellular domain (NICD) translocates into the nucleus and regulates gene expression of Hairy and Enhancer of Split (Hes) and Hairy-related (Hey) family members [74]. A great deal of research showed that Notch activation regulates angiogenesis through arterial/venous specification, angiogenic remodeling, and endothelial tip cell differentiation [75, 76]. One group [77] showed that the Notch ligand Jagged-1 (Jag1) present on endothelial cells is essential for neighboring vascular smooth muscle differentiation. Another group [78] demonstrated that the expression of Notch1 was increased after SCI and is upregulated when treated with drug. In this study, levels of Notch-1, Hes-1, and Jagged-1 proteins were significantly higher in the STS treated group compared to the SCI group 3 days after surgery. These findings indicated that the Notch signaling pathway plays an important role in improving angiogenesis. Moreover, the levels of Notch-1, Jagged-1, and Hes-1 proteins decreased after OGD exposure, which is reversed by STS, demonstrating that Notch signaling has protective effects on SCMECs *in vitro*. To further understand how the Notch pathway participated in this process, RO4929097, an inhibitor of the Notch signaling pathway used in human clinical trials [79], was studied to show that the protective effects of STS were reversed.

In conclusion, this study found that STS improves microcirculation both *in vitro* and *in vivo* through the activation of the Notch signaling pathway. These effects further exert neuroprotection in a SCI mouse model. This is the first report demonstrating that STS protects microcirculation in cases of SCI, indicating that STS may be a potential therapy.

## Data Availability

The data used to support the findings of this study are available from the corresponding author upon request.

## Abbreviations

BBB: Blood-brain barrier
BSCB: Blood spinal cord barrier
CNS: Central nervous system
EGM-2: Endothelial growth medium 2
FBS: Fetal bovine serum
HE: Histopathological evaluation
Hes: Hairy and Enhancer of Split
Hey: Hairy-related
NICD: Notch intracellular domain
OGD: Oxygen-glucose deprivation
PBS: Phosphate-buffered saline
PFA: Paraformaldehyde
SCI: Spinal cord injury
SCMECs: Spinal cord endothelial cells
STS: Sodium Tanshinone IIA silate
vWF: von Willebrand Factor
ZO-1: Zonula occludens-1.

## Supplementary Materials

Supplementary Materials The concise description for supplementary materials: Figure 1S: schematic diagram of the experimental protocols in vivo. As shown in this diagram, the different experiments were measured at different time points (1-28 days). The details of experiment methods refer to Materials and Methods.
