# Supplementary figures and images for: Sodium Tanshinone IIA Silate Exerts Microcirculation Protective Effects against Spinal Cord Injury In Vitro and In Vivo
Source: Oxid Med Cell Longev. 2020 Oct 8;2020:3949575. doi: 10.1155/2020/3949575 (PMC7568160; doi:10.1155/2020/3949575)

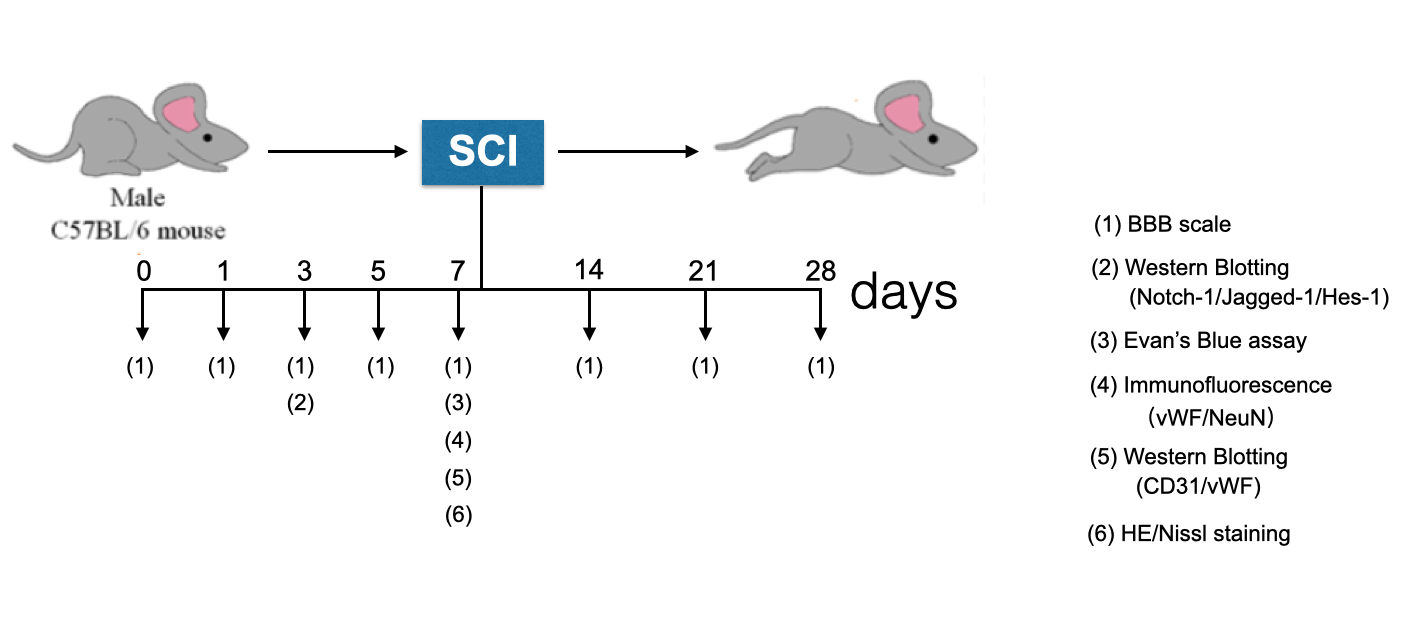

Supplement: Supplementary Materials — The concise description for supplementary materials: Figure 1S: schematic diagram of the experimental protocols in vivo. As shown in this diagram, the different experiments were measured at different time points (1-28 days). The details of experiment methods refer to Materials and Methods. [file 3949575.f1.tiff]
